# Lateral shoots removal has little effect on berry growth of grapevine (*Vitis vinifera* L.) ‘Riesling’ in cool climate

**DOI:** 10.1038/s41598-022-20246-z

**Published:** 2022-09-25

**Authors:** Qiuhong Ye, Hua Wang, Hua Li

**Affiliations:** College of Enology, Northwest A&F University, Yangling, 712100 China

**Keywords:** Physiology, Plant sciences

## Abstract

Bunch compactness is an important trait that affects the sanitary status and quality of wine grapes. Many studies have demonstrated that canopy managements, such as leaf removal, shoot trimming, and postponed first shoot topping, can effectively reduce compactness. However, few studies have determined the effects of canopy management measures on bunch compactness. Shoot wrapping has been previously shown to elongate the rachis length and reduce bunch compactness. Here, we tested whether the presence of laterals affects cluster growth in *Vitis vinifera* L. ‘Riesling’ through a field experiment with four treatments over two consecutive seasons: shoot wrapping with laterals, shoot wrapping without laterals, hedging with laterals, and hedging without laterals. Laterals were removed weekly. Lateral removal had little effect on cluster compactness; the effect was shown temporarily and not consistent the growing seasons. The effect of laterals on cluster compactness and rachis length slightly varied with years. The short-term and variable effect of laterals may be explained by the fact that they experienced little competition with clusters.

## Introduction

Bunch compactness is an important trait affecting the sanitary status and quality of wine grapes^1^. Compact bunches have higher incidences of pests and diseases^2,3^ for several reasons, including poor air circulation and limited sun exposure of the inner parts of the bunches ^2,4^, inefficient coverage of fungicide spray^5^, deficient development of epicuticular wax development of flattened berries^6,7^, and the burst of berries resulting from inner pressure^8^. The lack of sun irradiation received by interior berries results in inadequate phenolic maturation^9,10^.

OIV defined the bunch density by observation, like “1 = berries clearly separated, many visible pedicels”^11^. Bunch volume, bunch length, rachis length, and bunch weight are commonly used to objectively and quantitatively evaluate bunch compactness in research. The length of the internodes of the rachis has been noted to have a substantial effect on inflorescence openness^12^. Bunch compactness is affected by cultural practices as well as environmental and genetic factors, but the relative contributions of these different factors remain unclear^13^. Many agronomic practices have been used to obtain looser bunches. Some canopy management approaches are effective in reducing compactness, such as leaf removal^8,14,15^, shoot trimming^16^, and postponed first shoot topping^17^. However, the mechanisms by which these management approaches reduce cluster compactness have yet to be clarified. Bondada et al.^16^ cited various reasons why the effects of management approaches on compactness remain unclear. Hormones are known to regulate the growth and development of inflorescences in many ways. However, Grimplet et al.^18^ found that hormone concentrations (including indole-3-acetic acid, abscisic acid, jasmonic acid, salicylic acid, and gibberellic acids) did not differ between compact and loose clones and that there was no relationship between cluster compactness phenotype and auxin levels or auxin-related gene expression.

Previous studies have shown that the horizontal wrapping of shoots can promote increases in rachis length compared with hedging and sometimes result in reduced cluster compactness^19,20^. The wrapping of shoots can also reduce lateral emergence in the fruit zone^20,21^. To the best of our knowledge, few studies have determined why canopy management affects the rachis length or compactness. One hypothesis based on sink-source theory has been proposed to explain rachis elongation under shoot wrapping compared with hedging: hedging results in the rapid generation of large emerging laterals, and laterals compete with clusters for carbohydrates, which results in shorter rachis lengths of hedging vines. Although few studies have examined the effect of laterals on grapevine, most of these studies have focused on determining the effect of laterals on the fruit composition and yield components; by contrast, no studies have examined the effect of laterals on compactness or rachis length^22–24^. Here, a field experiment was conducted using *Vitis vinifera* L. cultivar Riesling, a typical compact cultivar, to determine the effect of laterals on compactness and rachis length.

## Results

### Bud survivability

There were no statistically significant differences between the no laterals treatment and control treatment in both years, regardless of the shoot tip treatment (Table 1). Bud survivability was significantly higher in 2019 than in 2018 in each treatment. The increase in bud survivability between years was higher in the no laterals treatments than in the treatments with laterals. Bud survivability of HL increased by 2.66% from 2018 to 2019, and bud survivability of HO increased by 4.31% from 2018 to 2019. The same pattern was observed in the shoot wrap treatments; SL and SO increased by 3.99 and 5.77% from 2018 to 2019, respectively.

**Table 1:** Effect of lateral removal on the bud survivability of ‘Riesling’.

Treatment	Bud survivability (%)		Percent change in bud survivability between years (%)
	2018	2019	
HL	83.93 ± 1.34	86.16 ± 1.37*	2.66
HO	86.23 ± 1.35	89.95 ± 1.37*	4.31
Sig	ns	ns	
SL	85.75 ± 1.04	89.17 ± 1.17*	3.99
SO	84.46 ± 1.04	89.33 ± 1.17*	5.77
Sig	ns	ns	

Significant differences between years (*p* < 0.05) are designated by *.

The phenological stage of flowering with 10% caps off (BBCH 61) was used as a comparison for annual plant phenology (Table 2). Lateral removal treatments each year are shown with date and BBCH.

**Table 2:** Treatment timing and key phenological growth stages in 2018 and 2019.

Year	BBCH	61	69	71	75	79	81
	Beginning of lateral removal	Flowering 10% caps off	Full bloom cap-fall complete	Setting	Berries pea size	Berries still hard and green	Veraison
2018	Jul 14 BBCH 75	Jun 15	Jun 28	Jul 1	Jul 10	Aug 8	Aug 12
2019	Jul 2 BBCH 68	Jun 21	Jul 3	Jul 5	Jul 15	Aug 6	Aug 17

### Berry growth

**Figure 1:**
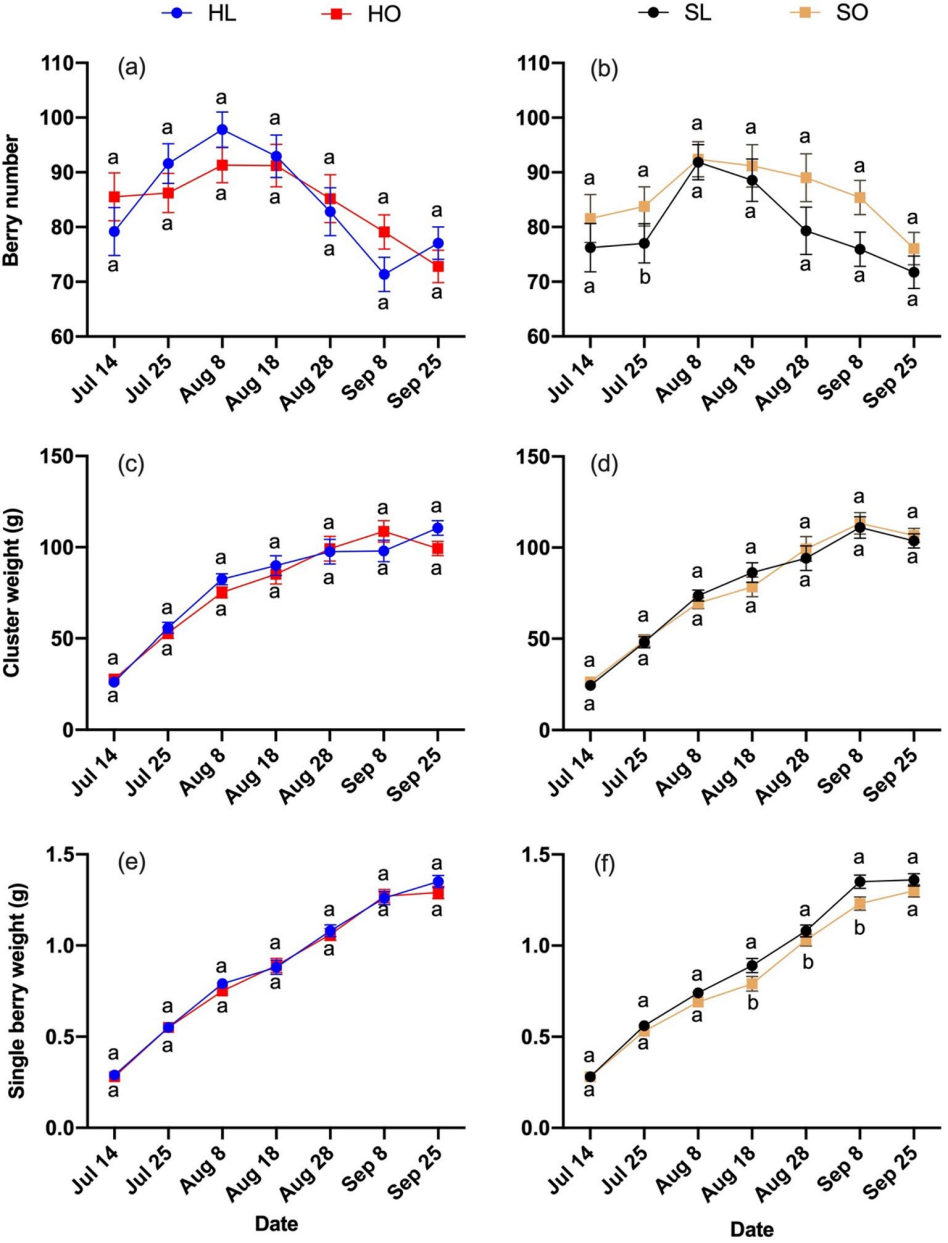
Berry weight traits in response to different treatments during the growing season in 2018 of ‘Riesling’. Berry weight traits: (**a**, **b**) Berry number, (**c**, **d**) Cluster weight, (**e**, **f**) Single berry weight. Treatments: (HL) Hedging with laterals, (HO) Hedging without laterals, (SL) Shoot wrap with laterals, (SO) Shoot wrap without laterals. Different lower-case letters showed significant differences at *P* ≤ 0.05 level.

**Figure 2:**
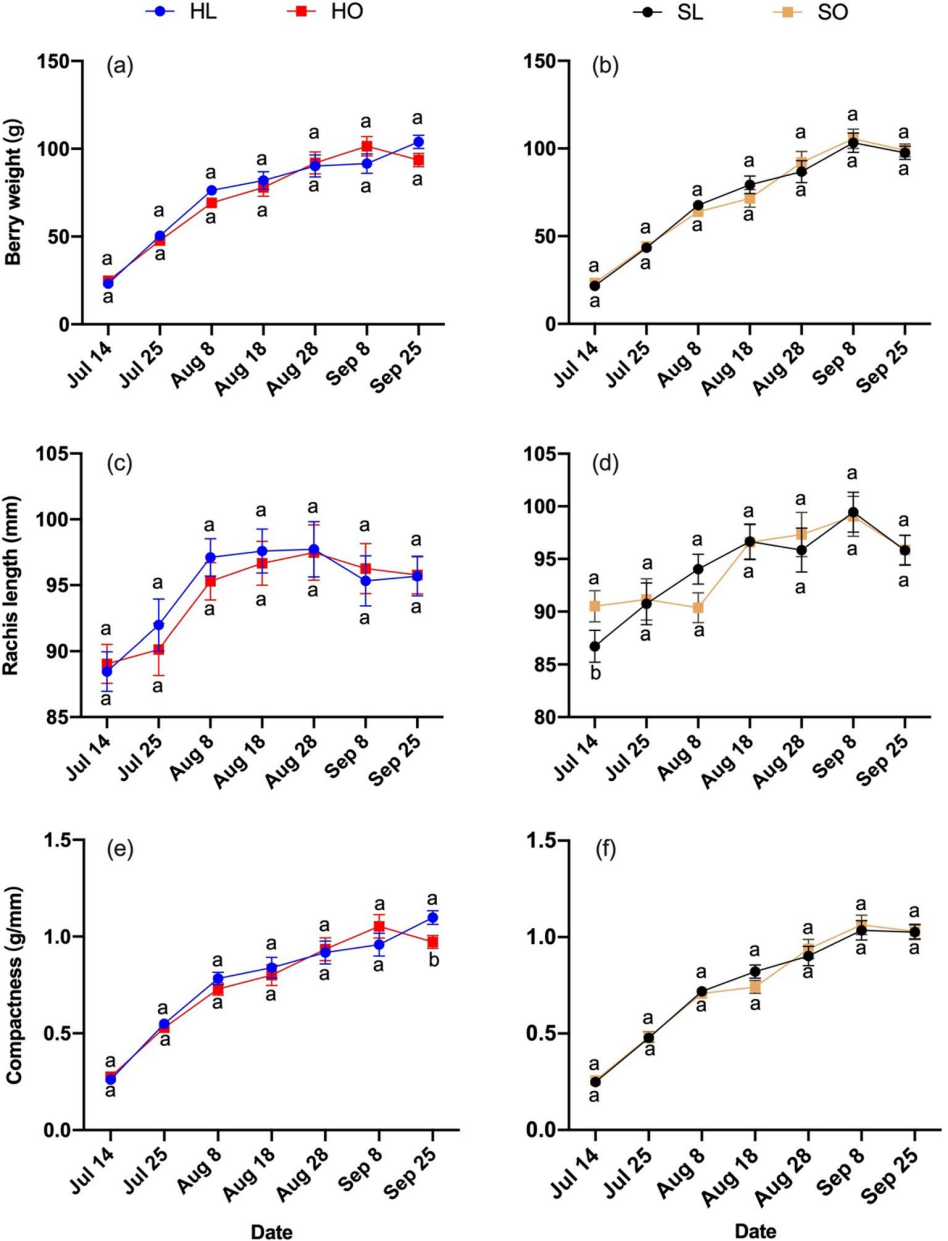
Berry compactness traits in response to different treatments during the growing season in 2018 of ‘Riesling’. Berry compactness traits: (**a**, **b**) Berry weight, (**c**, **d**) Rachis length, (**e**, **f**) Compactness. Treatments: (HL) Hedging with laterals, (HO) Hedging without laterals, (SL) Shoot wrap with laterals, (SO) Shoot wrap without laterals. Different lower-case letters showed significant differences at *P* ≤ 0.05 level.

**Figure 3:**
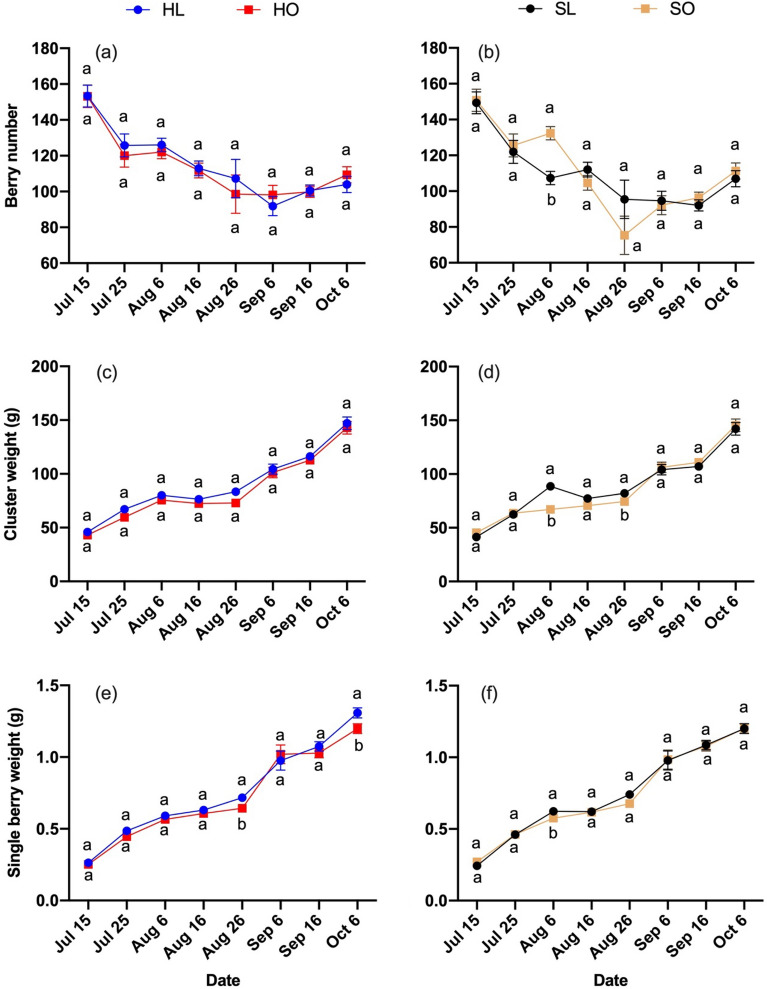
Berry weight traits in response to different treatments during the growing season in 2019 of ‘Riesling’. Berry weight traits: (**a**, **b**) Berry number, (**c**, **d**) Cluster weight, (**e**, **f**) Single berry weight. Treatments: (HL) Hedging with laterals, (HO) Hedging without laterals, (SL) Shoot wrap with laterals, (SO) Shoot wrap without laterals. Different lower-case letters showed significant differences at *P* ≤ 0.05 level.

**Figure 4:**
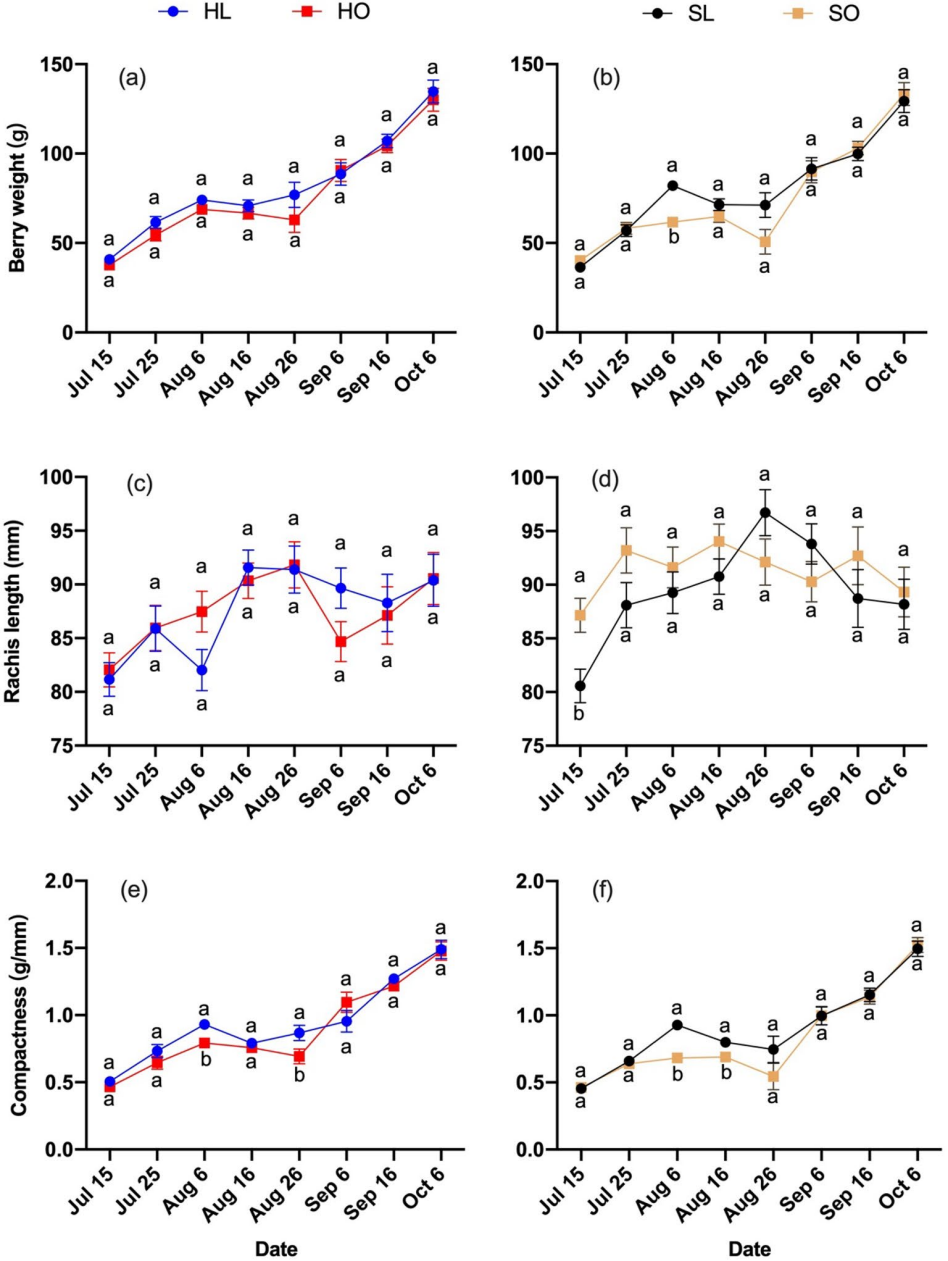
Berry compactness traits in response to different treatments during the growing season in 2019 of ‘Riesling’. Berry compactness traits: (**a**, **b**) Berry weight, (**c**, **d**) Rachis length, (**e**, **f**) Compactness. Treatments: (HL) Hedging with laterals, (HO) Hedging without laterals, (SL) Shoot wrap with laterals, (SO) Shoot wrap without laterals. Different lower-case letters showed significant differences at *P* ≤ 0.05 level.

Berry expansion in each treatment was basically similar during the sampling period in each season but differed between seasons (Figs. 1, 2, 3, 4). The accumulation of berry weight in 2019 generally followed an inverse sigmoidal pattern, whereas the accumulation of berry weight in 2018 resembled the last phase of a sigmoidal pattern. Changes in berry number differed in the two years. Berry number increased initially and then decreased at the end of 2018. Berry number decreased from the beginning and then did not change much during the last half of the sampling time in 2019. There was high variability in rachis length among samples, especially in 2019. There were no significant differences between HL and HO in berry weight and cluster weight in both years. The berry weight and cluster weight were twice significantly higher in SL than in SO in August 6, 2019 and August 26, 2019, respectively. No significant differences in berry weight and cluster weight were observed between SL and SO in 2018. Single berry weight varied more between treatments with laterals and without laterals. The single berry weight was higher in SL than in SO from August 8, 2018 to September 8, 2018. In 2019, the single berry weight was higher in SL than in SO on August 6, 2019. The single berry weight was higher in HL than in HO on August 26, 2019 and October 6, 2019. The berry number was higher in SO than in SL on July 25, 2018 and August 6, 2019. The rachis was longer in SO than in SL at the beginning of the sampling period in both years (July 14, 2018, BBCH 75 and July 15, 2019, BBCH 75). There was no difference between SL and SO in compactness during the growing season in 2018. Similar results were observed for HL and HO in 2018; the exception was that the compactness was higher in HL than in HO on September 25, 2018. Greater variation in compactness among treatments was observed in 2019. HL had higher compactness than HO on August 6, 2019 and August 26, 2019. Compactness was higher in SL than in SO on August 6, 2019 and August 16, 2019, which overlapped the period in which compactness differed between the hedging treatments.

### Shoot diameter

No differences were observed between treatments in shoot diameter during the growing season (Table 3, other data not shown). There were significant differences in shoot diameter between years for each treatment.

**Table 3:** Effect of lateral removal on the shoot diameter of ‘Riesling’.

Treatment	Diameter (mm)	
	Sep 6 2018	Sep 6 2019
HL	8.87 ± 0.19	8.89 ± 0.12
HO	8.94 ± 0.19	9.08 ± 0.12
Sig	ns	ns
SL	8.77 ± 0.19	8.72 ± 0.12
SO	8.69 ± 0.19	8.85 ± 0.12
Sig	ns	ns

### Laterals length

Sum of laterals length per shoot of Hedging and Shoot wrapping in 2018 were 24.04, 16.59 cm, respectively; in 2019 were 21.68 and 15.41 cm, respectively (Table S1). The laterals were short at first and second node (the first node was the node near the cane) compared to other nodes (Table S2).

### Yield components

There were no differences between treatments in yield or cluster number per vine (Table 4); however, there were significant differences in these traits between years for each treatment.

**Table 4:** Effect of lateral removal on the yield of ‘Riesling’.

Treatment	Year	Yield (kg)/v	Cluster number/v
HL	2018	4.49 ± 0.39	45.50 ± 2.29
HO		4.44 ± 0.39	44.58 ± 2.29
Sig		ns	ns
SL		4.32 ± 0.39	48.31 ± 2.29
SO		5.31 ± 0.39	47.17 ± 2.29
Sig		ns	ns
HL	2019	7.64 ± 0.44*	68.51 ± 3.26*
HO		6.49 ± 0.44*	68.76 ± 3.26*
Sig		ns	ns
SL		6.08 ± 0.44*	62.13 ± 3.26*
SO		6.42 ± 0.44*	59.89 ± 3.26*
Sig		ns	ns

Significant differences between years (*p* < 0.05) are designated by *.

### Fruit composition

The no laterals treatments had little effect on fruit composition. There was no significant difference in the soluble solids, titratable acidity, primary amino acids (PAA), ammonium, and YAN in both years (Table 5). The pH was higher in HL than in HO in 2018.

**Table 5:** Effect of lateral removal on the fruit composition of ‘Riesling’.

Treatment	Year	Brix	pH	TA	YAN
HL	2018	18.31 ± 0.24	3.37 ± 0.02a	6.67 ± 0.15	82.97 ± 10.33
HO		18.31 ± 0.24	3.29 ± 0.02b	6.34 ± 0.15	74.15 ± 10.33
Sig		ns	0.034	ns	ns
SL		18.22 ± 0.24	3.37 ± 0.02a	6.21 ± 0.15	83.07 ± 8.52
SO		18.26 ± 0.23	3.35 ± 0.02b	6.32 ± 0.15	68.65 ± 8.52
Sig		ns	ns	ns	ns
HL	2019	15.47 ± 0.22*	2.91 ± 0.01*	11.08 ± 0.35*	79.00 ± 8.06*
HO		15.52 ± 0.22*	2.89 ± 0.01*	10.93 ± 0.35*	64.17 ± 8.06*
Sig		ns	ns	ns	ns
SL		15.60 ± 0.29*	2.89 ± 0.01*	11.05 ± 0.35*	75.17 ± 8.42*
SO		15.23 ± 0.29*	2.88 ± 0.01*	11.13 ± 0.35*	74.00 ± 8.42*
Sig		ns	ns	ns	ns

Significant differences between treatments (*p* < 0.05) are designated by different letters. Significant differences between years (*p* < 0.05) are designated by *.

### Disease severity

The occurrence of leaf and fruit disease in each treatment was evaluated at the end of the season before harvest at BBCH 89. The most common leaf disease observed was downy mildew, and the most common insect pests observed were Japanese beetles. Fruit was mainly infected by sour rot. Sour rot was severe in 2018; the fruit disease index was as high as 55.17, and the fruit disease incidence rate reached 100% (Table 6). After implementing strict disease control measures in 2019, the fruit disease index decreased to 0.83, and the fruit disease incidence rate decreased to 24.63%. Leaf disease was not serious and was not evaluated in 2018. Leaves had a high disease incidence rate but low disease index; that is, most leaves were infected, but symptoms were light. The fruit disease index was significantly lower in HO than in HL in 2018. Because of severe sour rot in 2018, there were no differences in disease incidence between treatments with laterals and without laterals. There was light disease on fruit and leaves in 2019. There were no differences between treatments with and without laterals.

**Table 6:** Effect of lateral removal on the disease index and disease incidence rate of berries and leaves.

Treatment	2018		2019			
	Berry DI	Berry disease incidence rate (%)	Berry DI	Leaf DI	Berry disease incidence rate (%)	Leaf disease incidence rate (%)
HL	55.17 ± 5.37a	100.00 ± 2.18	0.83 ± 0.18*	5.90 ± 1.50	24.63 ± 4.12*	98.57 ± 0.88
HO	32.98 ± 4.73b	97.22 ± 1.96	0.28 ± 0.18*	7.43 ± 1.50	17.47 ± 4.12*	99.33 ± 0.88
Sig	0.023	ns	ns	ns	ns	ns
SL	47.52 ± 5.57	99.58 ± 1.96	0.42 ± 0.22*	4.17 ± 1.50	17.56 ± 4.12*	98.57 ± 0.88
SO	37.25 ± 5.56	97.50 ± 1.44	0.59 ± 0.22*	4.16 ± 1.50	20.72 ± 4.12*	98.58 ± 0.88
Sig	ns	ns	ns	ns	ns	ns

Significant differences between treatments (*p* < 0.05) are designated by different letters. Significant differences between years (*p* < 0.05) are designated by *.

## Discussion

The aim of this study was to assess the role that laterals play in cluster growth under shoot tip management. Our hypothesis was that laterals would compete with clusters for assimilates. When shoot tips were wrapped, lateral removal treatments promoted the elongation of rachis length; however, this effect did not persist in the presence of other disturbances (Figs. 2d, 4d). When shoot tips were hedged, lateral removal did not affect the rachis length in both years (Figs. 2c, 4c). Hedging is a major stimulation to vines compared with shoot wrapping. The effect of lateral removal was weakened in the presence of a more drastic stimulation such as hedging.

Although lateral removal is a common canopy management measure implemented in vineyards, few studies have examined the effect of lateral removal on grapevines. Several related studies found that the effect of lateral removal on berry quality might be limited and have little effect on yield or fruit composition. Candolfi-Vasconcelos^22^ implemented several types of canopy management schemes on Pinot noir, including comparing lateral removal with shoot topping (laterals retained). Lateral removal began six weeks after full bloom. No differences in yield were observed during the five-year experiment; however, yield was higher in treatments in which the laterals were retained than in treatments without laterals in the fourth year. The same was the case for the soluble solids, except that treatments in which laterals were retained had higher brix than treatments without laterals in the second year. No differences were observed in the acid content in the first three years (acid content data from the last two years were not collected). Lateral removal had no effect on the yield of Pinot noir in two consecutive seasons^23^. The laterals were removed starting at the full bloom stage. Lateral removal decreased the brix compared with the treatment in which laterals were retained but had no effect on the acid content. In a one-year study on ‘Riesling’ grapevines, the removal of laterals had no effect on the sugar concentration but decreased the acid concentration^24^. Laterals were removed three times during the growing season in this study. The effect of lateral removal on fruit composition and yield differed among these three studies. These differences might be explained by the fact that these studies were conducted in different locations with different climates or by the timing and frequency of lateral removal. Nevertheless, the precise cause of the differences between studies is difficult to determine.

In our study, lateral removal had little effect on the yield or fruit composition. Lateral removal had no effect on yield across the two years, which is consistent with the results of Candolfi-Vasconcelos^22^ and Vasconcelos and Castagnoli^23^. There was no effect of lateral removal on brix or acid concentration. Similar results were obtained in Candolfi-Vasconcelos, Carmo^22^, Vasconcelos and Castagnoli^23^, and Lampir^24^. Features of the training system and environment might explain variation in yield and fruit composition. Vines might have enough leaves for photosynthesis to sustain reproductive growth when laterals are removed. In the hedging without laterals treatments, which had the fewest leaves, each vine had 25 shoots and around 12 leaves per shoot. Smart and Robinson^25^ described the “ideal” shoot to be 2–3 feet long with 10–15 full-sized leaves. Thus, the vines in the experiment likely had a sufficient number of leaves to ensure high yield and maturity were attained. Even if there is a temporary shortage of assimilates in vines induced by lateral removal, they can compensate by improving their photosynthetic capacity^26,27^ and delaying leaf senescence and abscission^22^. Vines can also recover quickly with long days of abundant sunlight. Once the vines provide sufficient photosynthates for fruit, which is the main sink for photosynthates during the ripening phase, the surplus can be stored in reserves^28^. These possibilities might explain why lateral removal consistently had a negligible effect on yield and fruit composition.

Kaya^29^ compared the bud death of ’Bronx Seedless’, ’Cardinal’, ’Autumn Royal’, and ’Superior Seedless’ and showed that bud death was increased for buds in nodes with lateral shoots compared with buds in nodes without lateral shoots, suggesting that the presence of lateral shoots reduces the resistance of buds to low temperatures. The mechanism by which lateral shoots affect the cold hardiness of dormant buds of different grapevine cultivars remains unclear. Although we did not focus on the resistance of buds to cold hardiness, we determined bud survivability. The pattern of bud survivability in our study differed from that documented in Kaya^29^. There was no difference in bud survivability between treatments with and without laterals. Furthermore, bud survivability tended to be higher in treatments with laterals based on the percent change in bud survivability between years. The abundance of the second crop on the retained laterals was much higher (data not shown); this suggests that they might acquire more assimilates and compete against dormant buds with laterals nearby.

Cluster compactness has been shown to form via a complex and dynamic process over two years and depends on berry number, berry traits, and rachis traits^30,31^. These three components are determined at different stages of the grapevine reproductive cycle and are affected by many external factors such as temperature, rainfall, and wind^32,33^. These observations may explain why berry number, berry weight, and rachis length were affected by lateral removal at different times and for short periods. External factors such as temperature and rainfall may have a larger effect on cluster compactness than canopy management in some seasons.

Berry weight/rachis length and berry number/rachis length were used to evaluate compactness per Tello and Ibáñez^9^ and France^19^, respectively. In our study, several clusters had small berries. Although many of these were loose, the berry number was high. In addition, the pattern of berry number was unusual in 2018, and the pattern differed between years. Given these considerations, berry weight/rachis length was used to evaluate compactness.

Severe sour rot was prevalent at the end of the growing season of the experimental site in 2018, and this induced large losses of fruit. The fruit disease index and incidence rate reached up to 55.17 and 100%, respectively. Statistically significant differences in the fruit disease index were observed between HL and HO. Treatments without laterals tended to experience lighter symptoms than treatments with laterals. The resistance of fruit flies to pesticides was responsible for the severity of sour rot in the first year. Sour rot was effectively controlled in 2019 because a new pesticide was applied. Our results indicated that the efficiency of canopy management for the control of bunch rot varied depending on seasonal weather patterns and pesticide/fungicide management. This finding is similar to the patterns of leaf removal by botrytis rot observed by English et al.^34^.

The treatments were implemented during fruit set in the first year (Table 2) and then were applied to full bloom in the second year (Table 2). The little effect of lateral removal on berry growth was initially assumed to stem from the late timing of the treatment after the first growing season, which is why the treatment was applied earlier in the second year. However, the timing of the treatment does not appear to explain the small observed effect; alternatively, full bloom might not be a period when berry growth is greatly affected by lateral removal based on the second-year results. Coombe^35^ found that the removal of the apical part of the shoot (topping) increased set, but only if done when flower caps were falling. Additional treatment times need to be tested to determine if lateral removal has noticeable effects on berry growth.

The limited effect of lateral removal on berry growth could stem from seasonal weather patterns. Temperatures were generally higher in the growing season of 2018 than in 2019 (Table 7). There were fewer differences in 2018 than in 2019, which is consistent with the results of Frioni et al.^36^. Frioni et al.^36^ found that cluster thinning and leaf removal improved fruit composition at harvest in cooler summers, whereas no differences were found between treatments at harvest in warmer summers because the vines could develop efficiently through optimal temperature and light conditions. The efficacy of lateral removal might also be related to seasonal temperature patterns.

**Table 7:** Temperature and precipitation data of the experimental site during the growing seasons of 2018 and 2019.

Month	Year	Max Temp (℃)			Avg Temp (℃)			Min Temp (℃)				Precipitation (mm)			
		Max	Avg	Min	Max	Avg	Min	Max	Avg	Min	Max		Avg	Min	Sum
Apr	2018	21.67	8.74	− 0.56	13.96	3.58	− 4.07	7.78	− 2.09	− 17.78	15.24		1.27	0.00	39.62
	2019	23.33	13.72	2.22	14.65	7.22	− 2.74	8.89	1.17	− 17.78	22.35		1.78	0.00	54.61
May	2018	30.00	22.78	10.00	22.82	16.03	7.57	18.33	10.00	− 0.56	28.96		1.78	0.00	54.86
	2019	29.44	18.30	7.22	22.02	12.53	5.42	15.56	6.43	− 17.78	20.32		3.05	0.00	98.04
Jun	2018	33.89	23.82	15.56	25.81	17.73	11.23	20.00	10.94	− 17.78	12.45		1.02	0.00	27.18
	2019	30.00	23.21	15.00	23.03	17.37	10.42	17.22	11.26	2.78	23.11		3.56	0.00	109.98
Jul	2018	34.44	27.72	22.78	26.29	21.47	17.31	21.67	15.43	8.33	32.26		3.56	0.00	108.46
	2019	32.78	27.85	18.89	27.16	21.62	17.28	23.89	16.42	10.00	27.43		1.27	0.00	42.16
Aug	2018	32.22	26.14	21.11	26.29	21.05	15.96	22.22	16.90	8.89	24.64		2.54	0.00	79.25
	2019	29.44	25.25	18.89	23.67	18.93	13.03	19.44	13.55	7.22	20.07		2.29	0.00	67.06
Sep	2018	32.78	22.71	13.33	25.60	17.61	10.07	20.56	13.26	5.00	37.59		3.30	0.00	96.77
	2019	29.44	22.28	16.11	21.59	15.89	11.57	17.78	10.13	6.11	7.37		1.02	0.00	28.70
Oct	2018	26.67	13.64	4.44	22.38	9.33	0.87	19.44	4.77	− 17.78	20.07		2.79	0.00	87.88
	2019	29.44	16.20	7.22	22.09	10.65	5.99	16.67	5.81	− 2.22	23.37		2.03	0.00	66.80

The results of this study indicated that lateral removal had little effect on cluster compactness. The effect slightly varied with shoot tip treatments and depended on seasonal weather patterns. Additional treatment times need to be tested to confirm the effect of lateral removal on berry growth. Lateral removal had no effect on the yield and fruit composition in this study; however, additional research is needed to elucidate the long-term effects of lateral removal on vines. More canopy managements need to be explored to reduce cluster compactness to make berries healthier and better.

## Methods

### Experimental site and vines

The experiment was conducted at Cornell Orchards in Lansing, NY on the lower east side of Cayuga Lake (42.57°N, − 76.6°W, 124 m elevation). The experimental research and field studies on cultivated plants, including the collection of plant material, comply with relevant institutional, national, and international guidelines and legislation. The soil was mainly Hudson-Cayuga silt loam with 12–20% slopes^37^.

The vines were ‘Riesling’ cl.9/110 on 3309 rootstocks and were originally planted in 2007. There were 14 rows with 2.7 m × 1.8 m spacing, ten panels in each row, and four vines in each panel. Vines were trained in a two-tier flat bow system and vertical shoot positioned on two 2.4 m trellis with 0.98 m catch wire. Each vine was dormant pruned to four canes in late winter. Buds were counted from each vine after bud break; the same number of buds was then kept in each vine. Bud survivability was calculated by the alive buds/the whole buds each vine. Disease was controlled using standard practices for *V. vinifera* in the northeastern United States^38^. Key phenological growth stages was recorded according to BBCH system^39^.

Climate data for the site were recorded from the Cornell University Network for Environment and Weather Applications (NEWA) Lansing station (newa.cornell.edu), within 50 m of the research block.

### Experimental design

Four treatments were replicated six times in a randomized complete block design: Shoot wrap with laterals (SL), Shoot wrap—no laterals (SO), Hedging with laterals (HL), and Hedging—no laterals (HO). Diagrams of the shoot wrap management methods can be found in France^19^. The experimental block had 14 rows and 10 panels in each row. The two outer rows and two outer panels in each row were maintained as buffers and were not used for data collection. Each panel had four vines. Each treatment in each replication represented one experimental unit. The hedging, shoot wrapping and lateral removal were conducted when shoots’ height were 50 cm above the top wire at BBCH 75, BBCH 68 in 2018 and 2019, respectively. In the no laterals treatment, laterals were removed weekly as they arose.

### Cluster observation

One cluster sampled from each vine every sampling time. Clusters were collected randomly from 14 vines in each experimental unit every ten days (the two outer vines in each experimental unit were not used for data collection). Samples were brought to the lab in an ice container and kept in a 4 ℃ fridge. Rachis length, cluster weight, berry weight, and berry number were measured. Cluster weight and berries were weighed using a digital scale (0.01 g accuracy). Berry weight/rachis length was used to evaluate compactness. As we found the clusters have a complex and diverse structure, weight and rachis length provide limited information in the first year. Thus, we analyzed the shoulder (‘wing’/’outer arm’) in the second year.

### Primary shoot observation

Primary shoot diameter was measured every two weeks from bud break to the end of the season. Shoot diameter was measured at the internode between the second and the third nodes with digital calipers; the diameter of the shoot was measured at its largest and smallest points.

### Laterals observation

Four shoots each vine from two panels (eight vines) in the middle of each experimental unit were selected for laterals observation. Shoots were marked only for laterals observation and not sampled clusters. Laterals length of laterals at 1st to 12th node were measured by tapeline.

### Yield and composition analysis

Two panels (eight vines) in the middle of each experimental unit were harvested and weighed to determine the yield. Twenty clusters were sampled to determine the soluble solids (°brix), pH, titratable acidity (TA), yeast available nitrogen (YAN) from each experimental unit at harvest.

Soluble solids were measured using a digital refractometer with temperature compensation (Misco, model PA203X, Cleveland, OH), pH was measured using a calibrated pH meter (Fisher Scientific, Accument Basic AB15, Hampton, NH), and TA was measured by autotitrating 5 mL of juice with 0.10 M NaOH to a pH of 8.2 by a pH meter (Metrohm, 848 Titrino Plus, Switzerland). YAN was determined from juice samples by enzymatic analysis for primary amino nitrogen and ammonia (Randox Monaco RX, model RS-232, United Kingdom).

### Berry and leaf disease

Berry and leaf disease incidence and severity was assessed on 20 Sep. 2018 and 1 Oct. 2019, before harvest. Disease was evaluated in the middle of each data collection panel. Twenty leaves and ten fruits each experiment unit was randomly chosen for evaluation. The chosen were rated both for incidence (percentage of leaves having any sign of disease vs. percentage of leaves not having any sign) and for severity (11 ratings: 0%; 1–10%; 11–20%; 21–30%; 31–40%; 41–50%; 51–60%; 61–70%; 71–80%; 81–90%; 91–100% of leaf area infected). The severity evaluation was assisted by folding leaves to assess area.

### Statistical analysis

All data were analyzed in JMP Pro 14 (SAS Institute, Cary, NC) using a mixed-model ANOVA, with treatment as a fixed variable and block and experimental unit as random factors. Significance was determined using Tukey HSD at the 5% significance level. Significance of laterals length between treatments was analyzed by Wilcxon test as the data was abnormal distribution.

## Supplementary Information

Supplementary Information.
