# Supplementary material for: Lateral shoots removal has little effect on berry growth of grapevine (Vitis vinifera L.) ‘Riesling’ in cool climate
Source: Sci Rep. 2022 Sep 25;12:15980. doi: 10.1038/s41598-022-20246-z (PMC9510129; doi:10.1038/s41598-022-20246-z)
Supplement: Supplementary file 1 — Supplementary Information. [file 41598_2022_20246_MOESM1_ESM.docx]

Table S1. Lateral length on shoots of treatments with laterals in two years. Note: H: Hedging treatment; S: Shoot wrapping treatment (The same below).

| Year | Treatment | Shoot number | Sum of laterals length (cm) | Sum of laterals length per shoot (cm) | Difference by percent |
| --- | --- | --- | --- | --- | --- |
| 2018 | H | 188 | 4520.03 | 24.04 |  |
|  | S | 192 | 3185.20 | 16.59 | 44.93% |
| 2019 | H | 195 | 4226.75 | 21.68 |  |
|  | S | 201 | 3098.31 | 15.41 | 40.62% |

Table S2. Mean, standard error and median of lateral length (cm) on 1-12 node of treatments with laterals. Significant differences between treatments (p < 0.05) are designated by *.

| Node | Treatment | Aug 25 2018 | | | | Aug 25 2019 | | | | |
| --- | --- | --- | --- | --- | --- | --- | --- | --- | --- | --- |
|  |  | Mean | Std | Mdn | P value | Mean | Std | Mdn | | P value |
| 1 | H | 0.09 | 0.57 | 0 | 0.95 | 0.02 | 0.29 | | 0 | 0.19 |
|  | S | 0.05 | 0.34 | 0 |  | 0.06 | 0.43 | | 0 |  |
| 2 | H | 0.22 | 1.39 | 0 | 0.63 | 0.08 | 0.80 | | 0 | 0.45 |
|  | S | 0.29 | 2.04 | 0 |  | 0.03 | 0.16 | | 0 |  |
| 3 | H | 1.55 | 4.25 | 0.2 | 0.002* | 0.44 | 2.26 | | 0 | 0.81 |
|  | S | 0.82 | 2.36 | 0 |  | 0.34 | 1.23 | | 0 |  |
| 4 | H | 1.93 | 4.89 | 0.7 | 0.48 | 0.47 | 1.09 | | 0.1 | 0.64 |
|  | S | 1.59 | 4.98 | 0.8 |  | 0.46 | 0.87 | | 0.1 |  |
| 5 | H | 3.58 | 6.52 | 1.5 | 0.02* | 1.67 | 3.14 | | 0.5 | 0.98 |
|  | S | 2.79 | 6.25 | 1 |  | 1.92 | 4.30 | | 0.5 |  |
| 6 | H | 2.37 | 6.34 | 0.5 | 0.61 | 1.14 | 1.98 | | 0.2 | 0.31 |
|  | S | 1.59 | 4.19 | 0.5 |  | 0.80 | 1.18 | | 0.2 |  |
| 7 | H | 2.05 | 4.46 | 1 | 0.05 | 1.40 | 1.92 | | 0.8 | 0.009* |
|  | S | 1.34 | 2.65 | 0.5 |  | 1.38 | 3.42 | | 0.5 |  |
| 8 | H | 2.67 | 4.81 | 1.2 | 0.15 | 3.14 | 3.49 | | 2.5 | 0.11 |
|  | S | 1.90 | 3.30 | 1 |  | 2.57 | 2.67 | | 2 |  |
| 9 | H | 2.63 | 3.92 | 1.1 | 0.007* | 2.96 | 3.81 | | 2 | 0.007* |
|  | S | 1.98 | 4.46 | 0.8 |  | 2.22 | 2.97 | | 1.5 |  |
| 10 | H | 1.74 | 2.83 | 0.8 | 0.01* | 2.84 | 4.07 | | 1.5 | 0.003* |
|  | S | 0.99 | 1.75 | 0.5 |  | 1.62 | 2.24 | | 1 |  |
| 11 | H | 2.71 | 4.77 | 1.2 | 0.03* | 3.96 | 4.83 | | 2.5 | 0.003* |
|  | S | 1.84 | 3.04 | 1 |  | 2.38 | 2.85 | | 1.55 |  |
| 12 | H | 2.50 | 7.25 | 0.5 | 0.63 | 3.57 | 4.18 | | 2 | <.0001* |
|  | S | 1.39 | 2.16 | 0.5 |  | 1.67 | 2.71 | | 1 |  |
| Mean | H | 2.00 | 2.66 | 0.99 | 0.004* | 1.81 | 1.61 | | 1.35 | <.0001* |
|  | S | 1.38 | 1.59 | 0.75 |  | 1.28 | 1.26 | | 0.94 |  |
| Sum | H | 24.04 | 31.87 | 11.9 | 0.004* | 21.68 | 19.35 | | 16.2 | <.0001* |
|  | S | 16.59 | 19.12 | 9.05 |  | 15.41 | 15.08 | | 11.3 |  |
